# New Coordination Compounds Based on a Pyrazine Derivative: Design, Characterization, and Biological Study

**DOI:** 10.3390/molecules27113467

**Published:** 2022-05-27

**Authors:** Alina Climova, Ekaterina Pivovarova, Bartłomiej Rogalewicz, Anita Raducka, Małgorzata Szczesio, Izabela Korona-Głowniak, Agnieszka Korga-Plewko, Magdalena Iwan, Katarzyna Gobis, Agnieszka Czylkowska

**Affiliations:** 1 Institute of General and Ecological Chemistry, Faculty of Chemistry, Lodz University of Technology, Zeromskiego 116, 90-924 Lodz, Poland; ekaterina.pivovarova@dokt.p.lodz.pl (E.P.); bartlomiej.rogalewicz@dokt.p.lodz.pl (B.R.); anita.raducka@dokt.p.lodz.pl (A.R.); malgorzata.szczesio@p.lodz.pl (M.S.); 2 Department of Pharmaceutical Microbiology, Medical University of Lublin, 20-093 Lublin, Poland; izabela.korona-glowniak@umlub.pl; 3 Independent Medical Biology Unit, Medical University of Lublin, Jaczewskiego 8b, 20-090 Lublin, Poland; agnieszka.korga-plewko@umlub.pl; 4 Department of Toxicology, Medical University of Lublin, Chodzki 6, 20-093 Lublin, Poland; magda.iwan@umlub.pl; 5 Department of Organic Chemistry, Medical University of Gdansk, 107 Gen. Hallera Av., 80-438 Gdansk, Poland; katarzyna.gobis@gumed.edu.pl

**Keywords:** nickel(II), iron(III), cobalt(II), manganese(II), drug design, pyrazine derivative, anticancer, antibacterial, antifungal, U87 MG cancer cells, LN229 cancer cells

## Abstract

New coordination compounds of Mn(II), Fe(III), Co(II), and Ni(II) and the biologically active ligand L (*N*′-benzylidenepyrazine-2-carbohydrazonamide) were synthesized and characterized by appropriate analytical techniques: elemental analysis (EA), thermogravimetric analysis (TG–DTG), infrared spectroscopy (FTIR), and flame-atomic absorption spectrometry (F-AAS). The biological activity of the obtained compounds was then comprehensively investigated. Rational use of these compounds as potential drugs was proven by ADME analysis. All obtained compounds were screened in vitro for antibacterial, antifungal, and anticancer activities. Some of the studied complexes exhibited significantly higher activity than the ligand alone.

## 1. Introduction

Cancer is the general name for a large group of diseases that affect every part of the body. The terms “malignant tumors” and “neoplasms” are also used. The development of tumor is called carcinogenesis and refers to the transformation of normal tissue cells into cancerous cells. The fact is that the cells of human body divide not only at a young age during growth and development but throughout life, since live tissues are constantly renewed. According to the World Health Organization (WHO), cancer is the second leading cause of death in the world after cardiovascular disease. In 2020, WHO experts reported that 19.3 million people were diagnosed to have cancer. Ten million people died as a result of the disease [1]. Experts have emphasized that early diagnosis plays a key role in the treatment of cancer.

Based on the current situation, research works on anticancer drug development throughout the world have significantly increased. For more than a decade, coordination chemistry has been at the forefront in solving the problems presented above [2,3]. Coordination chemistry is a contemporary discipline of significantly increasing importance in both therapeutic and diagnostic medicine. Synthesized compounds containing fragments of organic ligands and metal ions are essential in targeted drug design. The main strategy of this branch of chemistry is the appropriate choice of the ligand and metal among the huge variety. The application of metal complexes has risen since last century. Metal-based complexes have attracted the attention of researchers because of their specific properties for cancer treatment. Complexes containing manganese ions have shown significant ability to be used as drugs [4,5,6]. Furthermore, the Mn(II) ion in a metal–ligand framework has found application as an inhibitor of MtDXR (*Mycobacterium tuberculosis*) [7,8,9]. Comprehensive studies have been conducted on Fe-based compounds, especially ferrocifen. This compound ought to be described as an organometallic derivative of a breast cancer drug. Therefore, ferrocifen derivatives have attracted considerable attention because of their antiproliferative activities and pharmacological profiles [10,11,12,13,14]. Cobalt has shown significant potential as an anticancer agent. Moreover, the metal center, positively charged Co(II), in addition to a stable coordinated structure plays essential roles in better DNA-targeting properties and reactive oxygen species (ROS) generation in mitochondria [15,16,17]. Expressive research has demonstrated the ability of various nickel-based complexes to be used as metal-based drugs with anticonvulsant, antibacterial, antifungal, antioxidant, and anticancer properties [18]. In addition, in coordination with amino acid Schiff bases, nickel complexes have demonstrated inhibitory properties against cancer cell growth, mainly through mitochondrial dysfunction and intracellular ROS accumulation [19,20].

**Figure 1:**
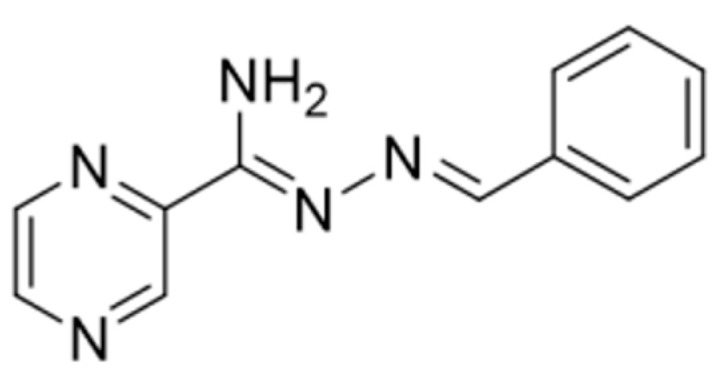
The structure of the organic ligand.

This research work was focused on the development of new coordination compounds with the pyrazine derivative L (*N*′-benzylidenepyrazine-2-carbohydrazonamide) as the organic ligand (Figure 1). Based on the analyzed literature, this type of organic ligand can be described as one of the most promising compounds with anticancer, antibacterial, and antifungal activities [21]. The imbalanced electron density distribution in the cycle is due to the strong inductive effect of two symmetrically arranged nitrogen atoms. Consequently, under normal conditions, no substitution at the carbon atoms occurs, while an attack at the same carbon atoms is possible. This significant chemical structure allows synthesizing a variety of corresponding derivatives that possess numerous pharmacological effects [22]. The metallic elements used in medicine have characteristic features crucial to their particular application and not shared with organic compounds. In order to understand why a particular metal should be chosen for a particular medical application, it is necessary to appreciate the properties and chemistry of the metal itself. Therefore, Mn(II), Fe(III), Co(II), and Ni(II) were chosen as the most promising and suitable candidates for cancer treatment. They were investigated because transition metal ions are small and polarizing, since their nuclei are poorly shielded. Therefore, d-block metals attract ligand strongly [23].

The aim of this work was the synthesis of new alternative coordination compounds to open possibilities for the pharmaceutical industry. The synthesized compounds were characterized with the appropriate analytical techniques: elemental analysis (EA), atomic absorption spectrometry (AAS), Fourier transform infrared spectroscopy (FTIR), and thermogravimetric analysis (TGA). In order to predict their biological properties, ADME analysis was performed. The presented in vitro investigations concentrated on the cell line L229 and U87 MG cells, with human skin fibroblast BJ (ATCC, Manassas, VA, USA) as a control normal cell line. This investigation also demonstrated the influence of the presence of transition metals on the antifungal and antibacterial properties of the ligand.

## 2. Results and Discussion

### 2.1. Activity Predictions

The ligand was tested in silico for antitumor activity (Table 1). The probability of activity towards oligodendroglioma was over 45%, and that towards pancreatic carcinoma, over 40%. Additionally, the compound was analyzed for its activity.

Activity was predicted for the obtained ligand L (Table 2). This compound was designed for antituberculosis activity. The aim of the present work was to test its applicability in the case of infections, during the treatment of tuberculosis, with other bacteria.

### 2.2. Thermogravimetric Analysis

**Figure 2:**
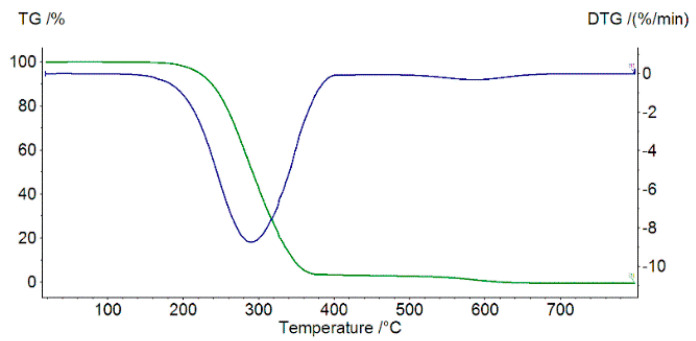
TG (green) and DTG (blue) curves of ligand decomposition in air.

**Figure 3:**
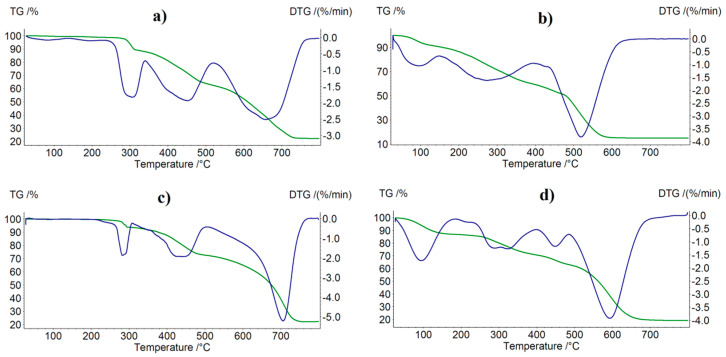
TG (green) and DTG (blue) curves of complexes decomposition in air: (**a**) Mn(L)Cl_2_, (**b**) Fe(L)Cl_3_∙CH_3_OH, (**c**) Co(L)Cl_2_, (**d**) Ni(L)Cl_2_∙C_2_H_5_OH.

Figure 2 and Figure 3 present thermal decomposition patterns of the free ligand and its Mn(II), Fe(III), Co(II), and Ni(II) complexes. All investigated compounds were stable at room temperature and, when heated, decomposed gradually. In the case of the coordination compounds, the final solid products of decomposition were M_3_O_4_-type metal oxides. Thermal decomposition data calculated or derived from decomposition patterns are presented in Table 3. In the case of the decomposition of Fe(L)Cl_3_∙CH_3_OH and Ni(L)Cl_2_∙C_2_H_5_OH complexes, the first step was the loss of the methanol or ethanol molecule, respectively. In the case of the complexes that did not contain alcohol molecules (Mn(L)Cl_2_, Co(L)Cl_2_), complexation significantly increased their thermal stability.

### 2.3. FTIR Spectra Analysis

**Figure 4:**
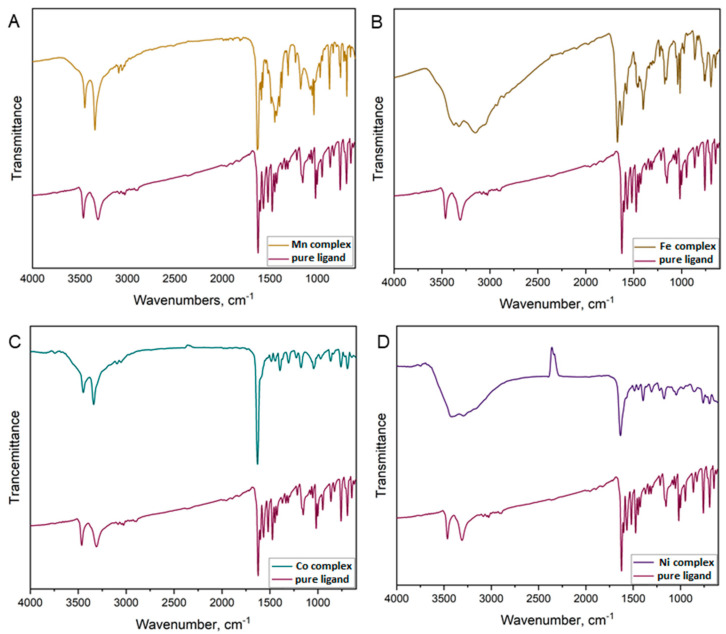
FTIR spectra of pure ligand and obtained complexes of (**A**) Mn(II); (**B**) Fe(III); (**C**) Co(II); and (**D**) Ni(II).

Figure 4 demonstrates the FTIR spectra of the pure ligand and its metal complexes. The spectrum of the pure ligand was compared with the spectra of the coordinated ligand with the d*^n^*-transition metals Mn(II), Fe(III), Co(II), and Ni(II). The spectrum of the uncoordinated ligand consisted of two bands at 3448 cm^−1^ and 3310 cm^−1^, which correspond to stretching vibrations of the NH_2_ group. In the case of the Mn(II) and Co(II) complexes, these two bands were moved because of the presence of an amino group, which did not take part in the process of complex formation. Coordination compounds of Fe(III) and Ni(II) had strong, broad peaks in the region of 3650–3200 cm^−1^, which were assigned to ν(O-H) stretching vibrations due to the presence of H_2_O molecules.

The strong absorption peaks observed in the pure ligand in the regions 1625–1600 cm^−1^ and 1474–1426 cm^−1^ were attributed to the ν(C=N) and ν(C=C) vibration modes. In all complexes, these bands were in the regions of 1670–1625 cm^−1^ and 1486–1450 cm^−1^, respectively. These modes shifted towards the higher wavenumber region after complex formation, which confirmed the involvement of the nitrogen atom in bonding with metal ions. Moving towards the lower wavelengths, we observed bands in the ranges 1394–1306 cm^−1^ and 970–690 cm^−1^ that corresponded to the β(CH) and γ(CH) modes, respectively. Their intensities were lower than those in the free ligand as a result of the coordination of the metal (II) ion with two nitrogen atoms. In the range 1220–1020 cm^−1^ appeared bands attributed to ν(C–N), belonging to alkylamines. In the case of coordinated ligands, these peaks were shifted to lower frequencies (1230–1040 cm^−1^). From the obtained spectra, it was clear that the ligand behaved as a bidentate ligand. The azomethine nitrogen atom and the nitrogen atom from the pyrazine ring were involved in metal(II) coordination.

### 2.4. ADME Analysis

**Figure 5:**
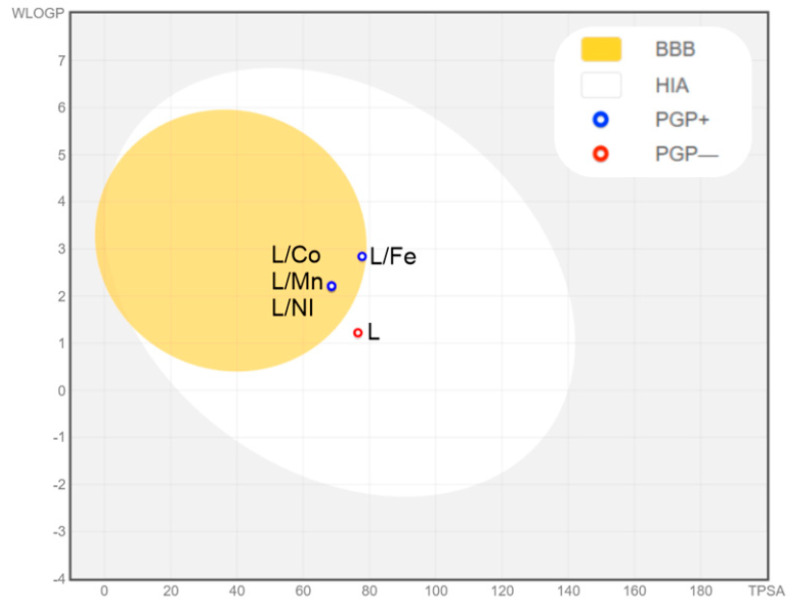
BOILED-Egg diagram for the ligand and obtained metal complexes. BBB (blood–brain barrier); HIA (passive gastrointestinal absorption).

The ADME analysis confirmed the effectiveness of the tested compounds crossing the blood–brain barrier, except for the pure ligand (Figure 5). The ligand was not a P-gp substrate, making it a good candidate against multidrug-resistant cancer cells. This compound met the rules of Lipinski, Ghose, Egan, Vebe, and Muegge [24,25,26,27,28]. Servis ProTox II classified the ligand into toxicity class 4 (predicted LD_50_: 400 mg/kg) and the complexes into toxicity class 3 (predicted LD_50_: 200 mg/kg) for the Ni(II) complex, toxicity class 4 (predicted LD_50_: 400 mg/kg) for Co(II) complex, and toxicity class 4 (predicted LD_50_: 1000 mg/kg) for the Mn(II) complex and Fe(III) complexes.

### 2.5. Antibacterial and Antifungal Activity

We screened the free ligand and its four complexes for activity against Gram-positive and Gram-negative bacteria, as well as against yeasts. Vancomycin (Van), ciprofloxacin (Cip), and nystatin (Nys) were used as the standard drugs (Table 4).

The tested compounds showed no activity against reference Gram-negative bacteria (*S.* Typhimurium, *E. coli*, *P. mirabilis*, *K pneumoniae*, and *P. aeruginosa*), with MIC > 1000 mg/L, except for Mn(L)Cl_2_, which had mild bioactivity (MIC range 500–1000 mg/L). Moderate bioactivity (MIC range 125–250 mg/L) was exhibited against *S. aureus S. epidermidis*, *M. luteus*, *E. faecalis*, *B. subtilis*, and *B. cereus* by the free ligand and against *M. luteus*, *E. faecalis*, and *B. subtilis* by the Mn(L)Cl_2_ compound. The compound Fe(L)Cl_3_∙CH_3_OH had very strong selective, bacteriostatic activity against staphylococci (*S. aureus*, *S. epidermidis*), and the Co(L)Cl_2_ complex was less active but also revealed good bioactivity against Gram-positive cocci (Table 4). All compounds were nonactive for reference strains of *C. albicans, C. parapsilosis*, and *C. glabrata* except Mn(L)Cl_2_, which showed moderate activity against yeast (126–500 mg/L). The lowest value (MIC = 250 mg/L) was obtained for the Mn(II) complex against *C. parapsilosis*.

### 2.6. Cytotoxicity Assay

**Figure 6:**
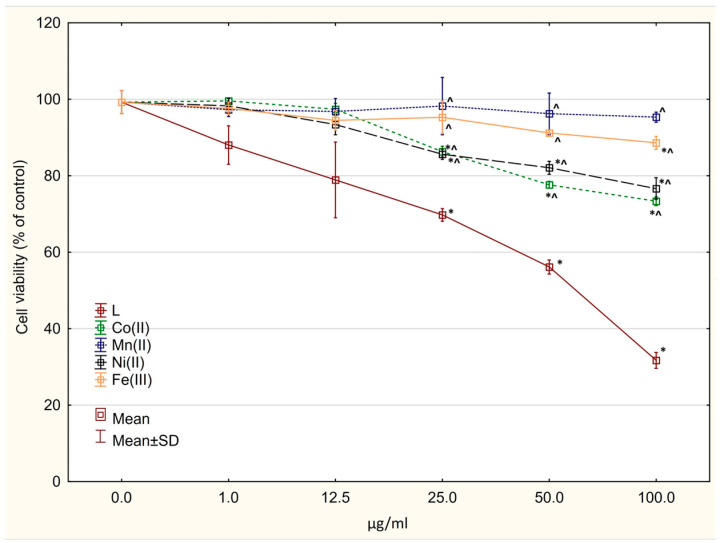
Cytotoxic activity of the investigated complexes and the ligand alone against BJ human normal cells. * *p* < 0.05 vs. control culture; ^ *p* < 0.05 vs. ligand (in corresponding concentrations).

**Figure 7:**
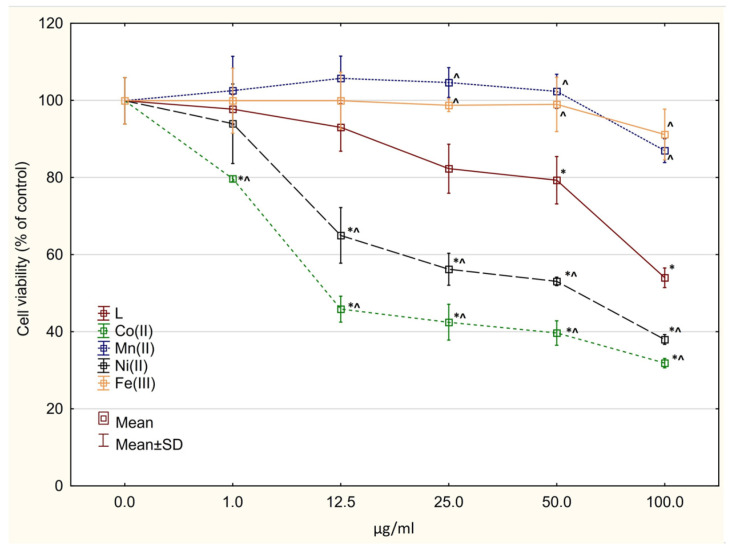
Cytotoxic activity of the investigated complexes and the ligand alone against U87 MG cancer cells. * *p* < 0.05 vs. control culture, ^ *p* < 0.05 vs. ligand (in corresponding concentrations).

**Figure 8:**
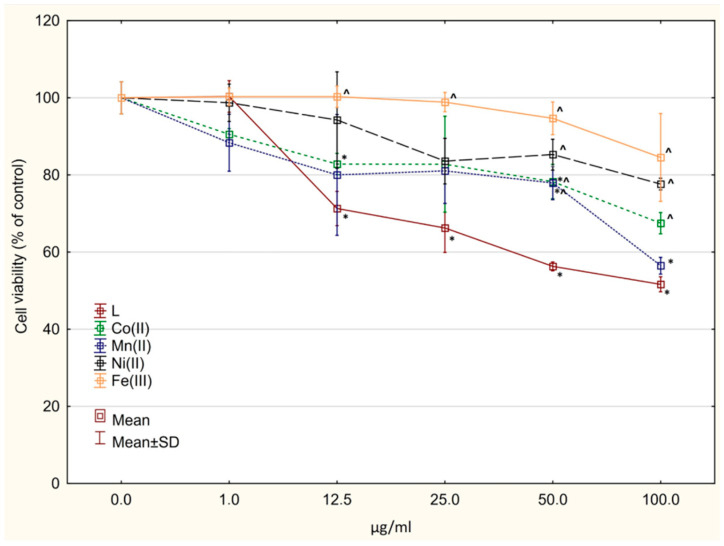
Cytotoxic activity of the investigated complexes and the ligand alone against LN229 cancer cells. * *p* < 0.05 vs. control culture, ^ *p* < 0.05 vs. ligand (in corresponding concentrations).

The free ligand showed cytotoxic activity against both normal (IC_50_ = 62.157 ± 3.58 µg/mL) and glioblastoma cells (IC_50_ ≈ 100 µg/mL for both cell lines) (Figure 6, Figure 7 and Figure 8). All tested complexes were slightly toxic to normal cells (IC_50_ > 100 µg/mL). A statistically significant decrease in cell viability was found for the compounds Co(L)Cl_2_ and Ni(L)Cl_2_∙C_2_H_5_OH starting at the concentration of 25 µg/mL (Figure 6). In all cases, complexation decreased the cytotoxic activity against normal cells (statistically significant in the range of concentrations 25–100 µg/mL, Figure 6). The compounds Co(L)Cl_2_ and Ni(L)Cl_2_∙C_2_H_5_OH revealed cytotoxicity against U87 MG cells (IC_50_ = 7.69 ± 2.17 and 42.82 ± 4.27 µg/mL, respectively) (Figure 7). The other complexes showed significantly lower activity in comparison with the ligand.

A significant decrease was also observed in the viability of LN229 cells after incubation with the compounds Mn(L)Cl_2_ and Co(L)Cl_2_ (Figure 8); however, the IC_50_ values were not reached in the range of concentrations used. The compound Fe(L)Cl_3_∙CH_3_OH showed weak activity on all three tested cell lines.

## 3. Materials and Methods

### 3.1. Chemistry

All of the chemicals used for the synthesis were purchased from Sigma-Aldrich, Alfa Aesar, and POCH and used without further purification. The contents of Mn(II), Fe(III), Co(II), and Ni(II) in solid complexes were determined by an F-AAS spectrometer with a continuum source of light and using air/acetylene flame. Absorbance was measured at analytical spectral lines; the limit of quantification was 0.04 mg/L. Solid samples were decomposed using an Anton Paar Multiwave 3000 closed system instrument. Mineralization was carried out for 45 min at 240 °C under a pressure of 60 bar. The contents of carbon, hydrogen, and nitrogen were determined by a vario MICRO Elementar Analysensysteme GmbH. The FTIR spectra were recorded with an IRTracer-100 Schimadzu Spectrometer (4000–600 cm^−1^) with an accuracy of recording of 1 cm^−1^ using KBr pellets. The thermolysis of the compounds in the air atmosphere was studied using the TG–DTG technique in the temperature range of 25–800 °C at a heating rate of 10 °C·min^−1^; TG and DTG curves were recorded on a Netzsch TG 209 apparatus under air atmosphere (v = 20 mL·min^−1^) using ceramic crucibles. Ceramic crucibles were also used as a reference material.

### 3.2. Synthesis of the Ligands

**Figure 9:**
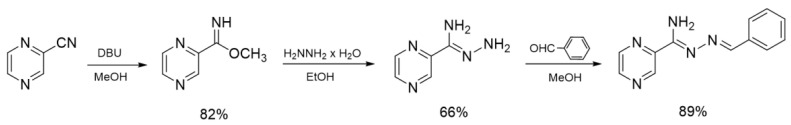
Synthesis route of the ligand.

The starting material for the synthesis was cyanopyrazine, which was converted into methyl pyrazine-2-carbimidate by the treatment of catalytic amounts of DBU in methanol. The iminoester, reacted with hydrazine hydrate, gave pyrazine-2-carbohydrazonamide. Next, amidrazone underwent a condensation reaction with benzaldehyde, which resulted in hydrazone L with a yield of 89% (Figure 9).

#### 3.2.1. Synthesis of Methyl Pyrazine-2-Carbimidate

First, 11 mL (0.12 mol) of cyanopyrazine was dissolved in 30 mL of MeOH, and 0.5 mL (3.3 mmol) of DBU (1,8-diazabicyclo [5.4.0]undec-7-ene) was added. The mixture was refluxed for 1 h and cooled, and the carbimidate precipitate was filtered off. Thus, 13.5 g (82%) of the product was obtained. Carbimidate was recrystallized from methanol. Analytical data were in accordance with the literature [29,30].

#### 3.2.2. Synthesis of Pyrazine-2-Carbohydrazonamide

First, 1.37 g (10 mmol) of the carbimidate was suspended in 5 mL of EtOH, and 1 mL of (32 mmol) 80% hydrazine hydrate was added. The mixture was refluxed for 15 min and then cooled. The carbohydrazonamide was isolated by filtration. Thus, 0.905 g (66%) of the product was obtained. Carbohydrazonamide was recrystallized from ethanol. Analytical data were in accordance with the literature [31,32].

#### 3.2.3. Synthesis of N’-Benzylidenepyrazine-2-Carbohydrazonamide (Ligand L)

First, 0.685 g (5 mmol) of carbohydrazonamide was dissolved in 15 mL of MeOH while hot, and 0.510 mL (5 mmol) of benzaldehyde was added. The mixture was left at room temperature for 0.5 h and then cooled, and the resulting imine was filtered off. Thus, 0.996 g (89%) of the product was obtained. The product was recrystallized from methanol. M.p. 161–164 °C; FTIR: 3465, 3312, 1625, 1598, 1568, 1520, 1475, 1449, 1167, 1154, 1019, 948, 158, 691, 648, 526, 503, 488 cm^−1^; ^1^H NMR (500 MHz, DMSO-d_6_): δ 7.15–7.30 (br s, 2H, NH_2_), 7.44–7.48 (m, 3H, Ph), 7.95–7.97 (m, 2H, Ph), 8.54 (s, 1H, CH), 8.74–8.75 (m, 1H, Pyr), 8.79 (d, 1H, Pyr, *J* = 2.7 Hz), 9.40 (d, 1H, Pyr, *J* = 1.4 Hz) ppm [32].

### 3.3. Synthesis of the Mn(II), Fe(III), Co(II) and Ni(II) Complexes

**Figure 10:**
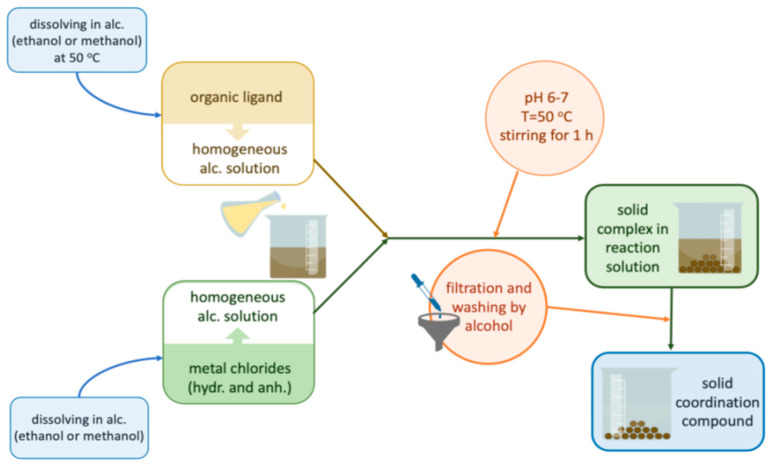
Synthesis route of the investigated complexes.

Figure 10 presents the synthesis route of the investigated complexes. The investigated Mn(II), Fe(III), Co(II), and Ni(II) complexes were synthesized using chloride salts of these metals. Mn(II), Co(II), and Ni(II) chlorides and the pure ligand were dissolved in ethanol at 50 °C using a magnetic stirrer. In the case of Fe(III) chloride, methanol was used as a solvent at the same temperature. The obtained ligand solutions were slowly poured under constant stirring into the solutions of metal chlorides (the molar ratio of the metal cations and the ligand in each case was 1:1) and left under stirring for 1 h at 50 °C. After that, the obtained mixtures were left in a refrigerator (4 °C) for 24 h. After that time, the obtained precipitates were filtered off and washed several times with the used solvents.

**Mn(L)Cl_2_ (C_12_H_11_N_5_MnCl_2_)** (351,12 g/mol), (yield 52%), anal. calculated (%): Mn, 15.65; C, 41.05; H, 3,16; N, 19.95. found (%): Mn, 15.58; C, 38.07; H, 2.93; N, 18.56. FTIR spectra (KBr, cm^−1^): ν(NH) 3448, 3342; ν(C=N) 1628; ν(C=C) 1485; δ(NH) 1447; β(CH) 1373, 1308; ν(C–N) 1175; ν(N-N) 1036; γ(CH) 872, 689.

**Fe(L)Cl_3_∙CH_3_OH (C_13_H_15_N_5_OFeCl_3_)** (419.53 g/mol), (yield 52%), anal. calculated (%): Fe, 13.31; C, 37.22; H, 3.61; N, 16.70. found (%): Fe, 11.12; C, 39.21; H, 3.53; N, 17.31. FTIR spectra (KBr, cm^−1^): ν(NH) 3374, 3323; ν(C=N) 1669; ν(C=C) 1462; δ(NH) 1445; β(CH) 1401, 1308; ν(C–N) 1176; ν(N-N) 1044; γ(CH) 862, 695.

**Co(L)Cl_2_ (C_12_H_11_N_5_CoCl_2_)** (355.11 g/mol), (yield 39%), anal. calculated (%): Co, 16.59; C, 40.58; H, 3.13; N, 19.73. found (%): Co, 16.91; C, 40.09; H, 3.13; N, 19.39. FTIR spectra (KBr, cm^−1^): ν(NH) 3447, 3339; ν(C=N) 1630; ν(C=C) 1485; δ(NH) 1445; β(CH) 1396, 1306; ν(C–N) 1176; ν(N-N) 1043; γ(CH) 886, 692.

**Ni(L)Cl_2_∙C_2_H_5_OH (C_14_H_17_N_5_ONiCl_2_)** (400.95 g/mol), (yield 31%), anal. calculated (%): Ni, 14.64; C, 41.94; H, 4.28; N, 17.47. found (%): Ni, 14.44; C, 41.13; H, 4.22; N, 17.00. FTIR spectra (KBr, cm^−1^): ν(NH) 3421, 3296; ν(C=N) 1636; ν(C=C) 1485; δ(NH) 1445; β(CH) 1394, 1306; ν(C–N) 1173; ν(N-N) 1045; γ(CH) 858, 694.

### 3.4. Activity Predictions

The ligand was analyzed using the service at www.way2drug.com/Cell-line accessed on 23 April 2022, a freely available web service for in silico prediction of human cell line cytotoxicity for druglike compounds [33]. The cell line cytotoxicity predictor (CLC-Pred) is a service for prediction of the cytotoxic effect of chemical compounds (http://www.way2drug.com/Cell-line) accessed on 23 April 2022 [34].

### 3.5. ADME Analysis

The ADME analysis was performed using the SwissADME service [35] (Swiss Institute of Bioinformatics 2021), a free web tool for evaluating the pharmacokinetics, druglikeness, and medicinal chemistry friendliness of small molecules (http://www.swissadme.ch/) (accessed on 23 April 2022); BOILED-Egg [36] to predict gastrointestinal absorption and brain penetration of small molecules; and the ProTOX II service [37] for prediction of the toxicity of the ligand.

### 3.6. Antibacterial and Antifungal Activity

The complexes were screened for antibacterial and antifungal activities by the microdilution broth method using Mueller–Hinton broth for growth of bacteria or Mueller–Hinton broth with 2% glucose for growth of fungi [38]. The minimal inhibitory concentrations (MIC) of the tested derivatives were evaluated for the panel of the reference microorganisms from the American Type Culture Collection (ATCC), including Gram-negative bacteria (*Escherichia coli* ATCC 25922, *Salmonella* Typhimurium ATCC 14028, *Klebsiella pneumoniae* ATCC 13883, *Pseudomonas aeruginosa* ATCC 9027, and *Proteus mirabilis* ATCC 12453), Gram-positive bacteria *(Staphylococcus aureus* ATCC 25923, *Staphylococcus aureus* ATCC 6538, *Enterococcus faecalis* ATCC 29212, *Micrococcus luteus* ATCC 10240, *Bacillus subtilis* ATCC 6633, and *Bacillus cereus* ATCC 10876) and fungi (*Candida albicans* ATCC 10231, *Candida parapsilosis* ATCC 22019, and *C. glabrata* ATCC 90030).

The complexes, dissolved in dimethyl sulfoxide (DMSO), were first diluted to the concentration (1000 mg/L) in an appropriate broth medium recommended for bacteria or yeasts. Then, using the same media, serial twofold dilutions were performed in order to obtain final concentrations of the tested derivatives ranging from 1.95 to 1000 mg/L. Sterile 96-well polystyrene microtitrate plates (Nunc, Roskilde, Denmark) were prepared by dispensing 200 µL of the appropriate dilution of the tested derivatives in broth medium per well. The inocula were prepared with fresh microbial cultures in sterile 0.85% NaCl to match the turbidity of 0.5 McFarland standard, and 2 µL was added to the wells to obtain final densities of 1.5 × 10^6^ CFU/mL for bacteria and 5 × 10^4^ CFU/mL for yeasts (CFU—colony-forming units). After incubation (35 °C for 24 h), the MICs were assessed visually as the lowest concentration of the extracts showing complete growth inhibition of the reference microbial strains. Appropriate DMSO control (at a final concentration <10%), a positive control (containing inoculum without the tested derivatives), and a negative control (containing the tested derivatives without inoculum) were included on each microplate.

Minimal bactericidal concentration (MBC) or minimal fungicidal concentration (MFC) was determined by subculturing 5 µL of the microbial culture from each well that showed growth inhibition, from the last positive culture, and from the growth control onto the recommended agar plates. The plates were incubated at 35 °C for 24 h, and the MBC/MFC was defined as the lowest concentration of the extracts without growth of microorganisms. Each experiment was repeated in triplicate. The highest MIC value was noted.

### 3.7. Cell Culture and Treatment

The studies were performed on two brain glioblastoma cell lines, U87 MG and LN229, and human skin fibroblast BJ (ATCC, Manassas, VA, USA) as a control normal cell line. LN229 cells were cultured in Dulbecco’s Modified Eagle’s Medium (Corning, New York City, NY, USA), and BJ and U87 MG cells were cultured in Eagle’s Minimum Essential Medium (EMEM) (Corning, New York City, NY, USA). The culture media were supplemented with 10% fetal bovine serum (PAN-Biotech, Aidenbach, Germany). The culture conditions were in accordance with the guidelines of ATCC for particular cell line. The cells were seeded into plates at a concentration of 1.5 × 10^5^ cells/mL and incubated at 37 °C with 5% CO_2_ air. When 70–80% culture confluence was achieved, the compounds were added. The cells were incubated for 48 h with the tested compounds in concentrations in the range of 1–100 µg/mL or with DMSO as a vehicle in control cultures (DMSO concentration in the cultures did not exceed <0.5%).

### 3.8. Cytotoxicity Evaluation

Cytotoxicity was evaluated with the MTT assay (ThermoFisher, Waltham, MA, USA). After 48 h of incubation with the compounds or DMSO, they were treated with MTT solution for 4 h. Obtained purple formazan crystals were dissolved in DMSO, and the resulting solution absorbance was measured using a PowerWave™ microplate spectrophotometer (Bio-Tek Instruments, Winooski, VT, USA) at a wavelength of 540 nm [39]. Each experiment was performed in triplicate with measurement in triplicate. The IC_50_ values were determined using the AAT Bioquest IC_50_ calculator (https://www.aatbio.com/tools/ic50-calculator) (accessed on 23 April 2022).

The results were statistically analyzed with Statistica v. 13 (StatSoft, Krakow, Poland). The data were expressed as mean ± SD. Statistical comparison of values was performed by one-way analysis of variance (ANOVA) and post hoc multiple comparisons with Tukey’s honestly significant difference (HSD) test. All parameters were considered statistically significantly different if *p* values were less than 0.05.

## 4. Conclusions

We present the method of synthesis of the *N*′-benzylidenepyrazine-2-carbohydrazonamide ligand and its four coordination compounds: Mn(L)Cl_2_, Fe(L)Cl_3_∙CH_3_OH, Co(L)Cl_2_ and Ni(L)Cl_2_∙C_2_H_5_OH. The ligand and all four complexes were stable at room temperature and, when heated, decomposed gradually. Investigations of the spectra of the obtained compounds suggested the bidentate manner of binding the metal centers via the azomethine nitrogen atom and the nitrogen atom from the pyrazine ring. The ADME analysis performed for both the ligand alone and its coordination compounds suggested potent biological activity. The synthesis of the complex was aimed at increasing the likelihood of crossing the blood–brain barrier. In silico research confirmed the assumptions about the anticancer effect. The glioblastoma lines were selected because the tested compounds showed a crossing of the blood–brain barrier and a high probability of being active against these tumor cells. The complexes Co(L)Cl_2_ and Ni(L)Cl_2_∙C_2_H_5_OH showed significant activity against U87 MG tumor cells (IC_50_ = 7.69 ± 2.17 and 42.82 ± 4.27 µg/mL respectively). It is also important to highlight that in all cases, complexation decreased cytotoxicity against normal cells. The free ligand exhibited moderate bioactivity against the reference Gram-positive cocci (*S. aureus*, *S. epidermidis*, *M. luteus*, *E. faecalis*) and Gram-positive, spore forming bacilli (*B. subtilis* and *B. cereus*). Even though the tested complexes did not exhibit significantly higher bioactivity, Fe(L)Cl_3_∙CH_3_OH showed very strong selective, bacteriostatic activity against staphylococci (*S. aureus*, *S. epidermidis*), and the Co(L)Cl_2_ complex was less active but had good bioactivity against Gram-positive cocci. Mn(L)Cl_2_ showed moderate activity against yeasts (126–500 mg/L). The improvement in the biological activity resulting from the complexation highlights the great possibilities of coordination chemistry in terms of possible applications in medicine.

## Figures and Tables

**Table 1:** Cancer cell line prediction results for the ligand. Pa (probability “to be active”); Pi (probability “to be inactive”).

Pa	Pi	Cell-Line Name	Tissue/Organ	Tumor Type
0.489	0.063	Oligodendroglioma	Brain	Glioma
0.417	0.072	Pancreatic carcinoma	Pancreas	Carcinoma
0.336	0.052	Hepatoblastoma	Liver	Hepatoblastoma
0.307	0.050	Renal carcinoma	Kidney	Carcinoma
0.294	0.085	Leukemic T-cells	Blood	Leukemia
0.303	0.135	Plasma cell myeloma	Blood	Myeloma

**Table 2:** Activity prediction results for the ligand. Pa (probability “to be active”); Pi (probability “to be inactive”).

Pa	Pi	Activity
0.675	0.011	HMGCS2 expression enhancer
0.652	0.005	Antituberculotic
0.651	0.007	Antimycobacterial
0.655	0.018	Pterin deaminase inhibitor
0.640	0.005	Antiprotozoal
0.627	0.006	Albendazole monooxygenase inhibitor
0.597	0.044	Mannotetraose 2-alpha-N-acetylglucosaminyltransferase inhibitor
0.592	0.040	Complement factor D inhibitor
0.569	0.023	Glutamine-phenylpyruvate transaminase inhibitor
0.590	0.048	Omptin inhibitor
0.573	0.035	Limulus clotting factor B inhibitor
0.600	0.070	Nicotinic alpha6beta3beta4alpha5 receptor antagonist
0.550	0.032	Antiviral (Picornavirus)

**Table 3:** Thermal decomposition data calculated or derived from decomposition patterns of the free ligand and its Mn(II), Fe(III), Co(II) and Ni(II) complexes.

Compound	Temperature Range (°C)	Mass Loss (%)		Intermediate Product or Solid Residue
		Found	Calculated	
Ligand	140–170	100.0	100.00	Total decomposition
Mn(L)Cl_2_	240–760	78.0	78.05	Mn_3_O_4_
Fe(L)Cl_3_∙CH_3_OH	40–140	8.5	7.64	Fe(L)Cl_3_
Fe(L)Cl_3_	140–650	75.5	73.96	Fe_3_O_4_
Co(L)Cl_2_	240–770	77.5	77.40	Co_3_O_4_
Ni(L)Cl_2_∙C_2_H_5_OH	40–160	12.5	11.49	Ni(L)Cl_2_
Ni(L)Cl_2_	160–740	68.0	68.55	Ni_3_O_4_

**Table 4:** Activity of the tested compounds against bacteria and yeasts presented as minimal inhibitory concentration values (mg/L).

Chemicals Microorganisms	L	Mn(L)Cl_2_	Fe(L)Cl_3_∙CH_3_OH	Co(L)Cl_2_	Ni(L)Cl_2_∙C_2_H_5_OH	Van
**Gram-Positive Bacteria**						
*S. aureus* ATCC 25923	250	1000	7.8	62.5	>1000	0.98
*S. epidermidis* ATCC 12228	125	500	7.8	500	>1000	0.98
*M. luteus* ATCC 10240	125	125	500	62.5	500	0.12
*E. faecalis* ATCC 29212	125	125	1000	500	1000	1.95
*B. subtilis* ATCC 6633	125	125	1000	500	1000	0.24
*B. cereus* ATCC 10876	250	500	1000	500	>1000	0.98
**Gram-negative bacteria**						**Cip**
*S.* Typhimurium ATCC 14028	>1000	500	>1000	1000	>1000	0.061
*E. coli* ATCC 25922	>1000	500	>1000	1000	>1000	0.015
*P. mirabilis* ATCC 12453	500	500	>1000	500	>1000	0.03
*K. pneumoniae* ATCC 13883	>1000	1000	>1000	1000	>1000	0.12
*P. aeruginosa* ATCC 9027	>1000	>1000	>1000	>1000	>1000	0.49
**Yeasts**						**Nys**
*C. albicans* ATCC 102231	1000	500	1000	>1000	>1000	0.48
*C. parapsilosis* ATCC 22019	1000	250	1000	>1000	1000	0.24
*C. glabrata* ATCC 90030	>1000	500	>1000	500	>1000	0.24

## Data Availability

Not applicable.

## Sample Availability

Samples of the compounds are available from the authors.
